# Supplementary material for: Recombinant Aflatoxin-Degrading F420H2-Dependent Reductase from Mycobacterium smegmatis Protects Mammalian Cells from Aflatoxin Toxicity
Source: Toxins (Basel). 2019 May 8;11(5):259. doi: 10.3390/toxins11050259 (PMC6563500; doi:10.3390/toxins11050259)
Supplement: Supplementary file 1 [file toxins-11-00259-s001.pdf]

Supplementary Materials: Recombinant Aflatoxin-Degrading $F_{420}H_2$-Dependent Reductase from *Mycobacterium smegmatis* Protects Mammalian Cells from Aflatoxin Toxicity

Che-Hsing Li, Wei-Yang Li, I-Ning Hsu [#], Yung-Yu Liao [#], Chi-Ya Yang [#], Matthew C. Taylor, Yu-Fan Liu, Wei-Hao Huang, Hsiang-Hua Chang, Ho-Lo Huang, Shao-Chi Lo, Ting-Yu Lin, Wei-Che Sun, Ya-Yi Chuang, Yu-Chieh Yang, Ru-Huei Fu * and Rong-Tzong Tsai *

# These authors contributed to the work equally and should be regarded as co-third authors.

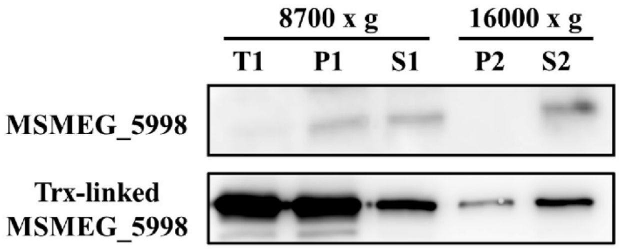

Figure S1. Relative protein concentration of affinity-purified native MSMEG_5998 and thioredoxin (Trx)-linked MSMEG_5998. The produced native MSMEG_5998 and Trx-linked MSMEG_5998 were purified by nickel-chelate affinity chromatography under native conditions. Subsequent samples from the purification process were examined by western blotting. *Escherichia coli* cells producing the proteins of interest were first broken by sonication (Section 5.3 in Materials and Methods), and total cell lysates (T1) were centrifuged. After the first centrifugation at 8700× *g*, total lysates were separated into a pellet (P1) and supernatant (S1). The supernatant was further separated into a pellet (P2) and supernatant (S2) by a second centrifugation, at 16,000× *g*. The preparation of T1, P1, S1, P2, and S2 fractions was described in Materials and Methods (Section 5.3). The fractions were adjusted to the same value. Because of the similar molecular weights of the proteins, the proteins were analyzed on individual SDS-PAGE gels. Native and Trx-linked MSMEG_5998 were detected by western blotting. Because of the similar molecular weights of the analyzed proteins, the proteins were analyzed on separate SDS-PAGE gels.

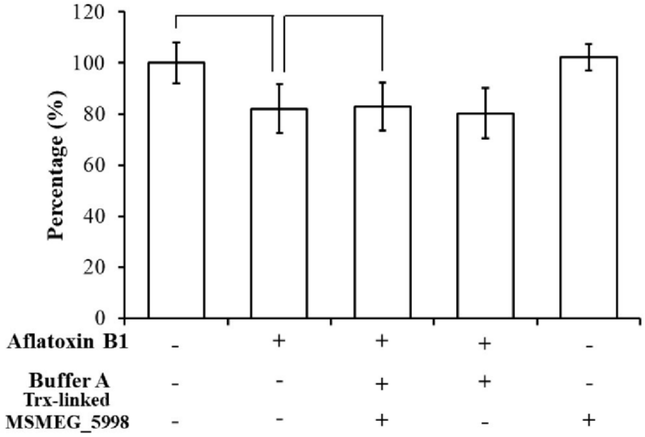

Figure S2. The effect of aflatoxin B1 (AFB1) and Trx-linked MSMEG_5998 on the viability of HepG2 cells. For the determinations, 1×10^4 cells/well in 96-well plates were treated, or not, with aflatoxin B1, buffer A, and Trx-linked MSMEG_5998 (the amounts of these chemicals were described in Section 5.5 in Materials and Methods) for 24 h. Then, cell viability was determined using the thiazolyl blue tetrazolium bromide (MTT) assay. No statistical differences were noted between the five groups.

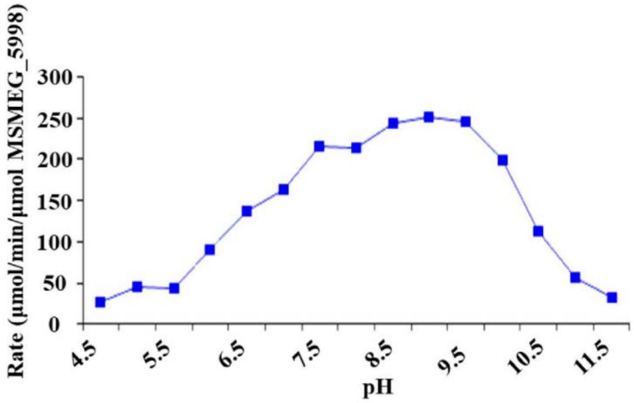

Figure S3. The pH dependence of the AFB1-degrading activity in native MSMEG_5998. The reaction rate of native MSMEG_5998 was measured using 20 mM buffer at varying pH, as reported by Beutler et al. [1]. The condition and process of enzyme activity measurement were described in Section 5.5 in Materials and Methods. The AFB1 concentrations were quantified through High Performance Liquid Chromatography.

References

1. Beutler, E.; Mitchell, M. Special modifications of the fluorescent screening method for glucose-6-phosphate dehydrogenase deficiency. *Blood* **1968**, *32*, 816–818.
